# Combined Occlusion Therapy and Home-Based Perceptual Learning in Children with Persistent Amblyopia: A Longitudinal Case Series

**DOI:** 10.3390/jcm15051817

**Published:** 2026-02-27

**Authors:** Maria Pérez-Benito, Raquel Amigo-Gamero, Teresa Calderón-González, Juan de la Cruz Cardona-Pérez, Santiago Martín-González, Juan A. Portela-Camino

**Affiliations:** 1 Servicio de Oftalmología, Hospital de Mérida, 06800 Mérida, Badajoz, Spain; mariaperezbe@salud-juntaex.es (M.P.-B.); raquelamigo2795@gmail.com (R.A.-G.); 2 Servicio de Oftalmología, Hospital Vital Álvarez Buylla, 33611 Mieres, Asturias, Spain; mariateresa.calderon@sespa.es; 3 Department of Optics, Instituto de Investigación Biosanitaria Ibs, University of Granada, 18012 Granada, Granada, Spain; cardona@ugr.es; 4 Department of Construction and Manufacturing Engineering, University of Oviedo, 33204 Gijon, Asturias, Spain; 5 Department of Optometry, Begira Ophthalmologic Clinic, 48001 Bilbao, Euskadi, Spain; juanportel@hotmail.com

**Keywords:** persistent amblyopia, perceptual learning, occlusion therapy

## Abstract

**Objectives**: Persistent amblyopia often shows limited response to occlusion therapy once visual acuity improvement plateaus. This study evaluated the efficacy of a two-phase protocol combining occlusion therapy and home-based perceptual learning (PL) in children with persistent amblyopia, including those with congenital pathology. **Methods**: This longitudinal case series included 40 patients (mean age 9.4 ± 3.4 years). Phase I consisted of occlusion therapy until best-corrected visual acuity (BCVA) plateaued. Phase II combined continued occlusion with home-based PL training until BCVA in amblyopic eye reached 0.00 logMAR or treatment was discontinued. BCVA and stereoacuity (TNO test) were assessed at baseline, after Phase I, after Phase II when applicable, and at a prospective evaluation visit. Treatment success was defined as a gain of ≥2 logMAR lines or a final BCVA ≤0.10 logMAR. Patients were stratified according to cumulative training exposure (<10 h vs. ≥10 h). **Results**: After Phase I, mean BCVA improved from 0.45 ± 0.23 to 0.26 ± 0.19 logMAR (*p* < 0.01). After Phase II, BCVA further improved to 0.13 ± 0.16 logMAR (*p* < 0.01). Stereoacuity showed a modest but significant improvement, from 928 ± 505 to 748 ± 558 arcsec (*p* = 0.01). Treatment success was achieved in 72% of patients completing ≥10 h of perceptual learning compared with 40% in those completing <10 h (RR = 1.94, 95% CI 1.01–3.73). Patients with non-pathological amblyopia achieved greater final BCVA than those with congenital pathology. **Conclusions**: The combination of occlusion therapy and home-based PL was associated with further improvement in visual acuity and modest gains in stereoacuity in children with persistent amblyopia. Greater cumulative training exposure was associated with higher treatment success, supporting PL as a clinically valuable adjunct to standard amblyopia management.

## 1. Introduction

Amblyopia is a common neurodevelopmental abnormality that results in physiological alterations in the visual pathways and impaired vision [1]. Amblyopia is associated with strabismus, anisometropia, or form deprivation and may affect one or both eyes [2]. Its prevalence in the general population is estimated to range from 1.3% to 3.6% [3]. In monocular amblyopia, the current standard of care includes optical correction of refractive error followed by penalization or direct occlusion of the fellow eye [4]. While 73% to 90% of patients achieve some improvement in visual acuity with conventional therapy, nearly half fail to reach normal visual acuity, and regression is common even in initially successful cases [5]. Buckle et al. [6] evaluated real-world outcomes of occlusion therapy in clinical settings, revealing that visual acuity gains plateaued after 32 weeks in mild cases and 48 weeks in severe cases.

Deprivation amblyopia results from any pathology that impedes the visual pathway, leading to partial or complete obstruction and degraded retinal image formation [7]. Common etiologies include congenital cataracts, ptosis, vitreous hemorrhage, corneal leukoma, and other structural anomalies [8]. Affected individuals often present with coexisting ocular conditions, such as microphthalmos, coloboma, optic nerve hypoplasia, or malformations [8]. Congenital or infantile cataract is the most frequent cause of deprivation amblyopia and often requires early surgical intervention [9]. However, the visual prognosis remains poor, particularly in unilateral cases, with high rates of severe amblyopia (~68%), strabismus (85%) [10,11,12], and an almost complete absence of measurable stereoacuity after treatment [13].

In order to improve the post-surgical visual acuity in congenital or infantile cataract eye, occlusion of the fellow eye is the gold standard treatment [8]. In these patients, compliance with occlusion therapy is notably poor due to severely reduced acuity in the amblyopic eye and associated frustration [13]. Even with rigorous occlusion regimens, fewer than one-third of patients attain a visual acuity better than 0.6 logMAR before the age of four [13]. Aggressive occlusion of the fellow eye has been proposed but is supported primarily by case series rather than randomized controlled trials [8]. Given these limitations, perceptual learning (PL) has emerged as a promising alternative. PL refers to relatively permanent and consistent changes in perceptual performance resulting from practice or repeated exposure to specific stimuli [14]. It involves targeted stimulation of cortical visual areas and promotes neuroplasticity [15,16]. The effectiveness of PL is dependent on stimulus richness, intensity, and the number of practice trials required for skill acquisition [17].

An increasing body of evidence supports PL as a viable therapeutic strategy in functional amblyopia. Multiple studies and systematic reviews have shown significant improvements in a wide range of visual functions in both pediatric and adult populations [15,16,18,19]. In amblyopia secondary to ocular pathology, PL has also yielded promising results, including in patients with central or peripheral field loss [20], cortical blindness, [21] and children with visual impairments [22,23]. Notably, Hamm et al. reported modest gains in visual acuity following a six-week dichoptic stimulation program in 18 patients with deprivation amblyopia due to congenital cataracts [24].

Among the tools employed in PL, Gabor patches (sinusoidal gratings enveloped by a Gaussian function) are widely used [18,19,25]. These stimuli, presented at varying spatial frequencies and contrasts, allow for training at or near the patient’s perceptual threshold. Improved detection of high-frequency Gabor stimuli has been shown to correlate with enhanced optotype visual acuity [1].

More recently, PL paradigms have been embedded in home-based video game formats. One such platform, Visionary (VisionaryTool LLC, Gijón, Spain; version VTV082025), integrates dichoptic Gabor stimuli in a gamified therapeutic environment. In a completed study, patients with patch-resistant amblyopia who received combined occlusion and home-based PL training showed significantly greater improvements than those treated with occlusion alone [26]. A recent case series has reported on patients with persistent esotropic amblyopia who received a combined treatment approach (occlusion therapy, in-office orthoptic training, and home-based PL therapy) with promising results in both visual acuity and stereoacuity [27].

Given this background, the present study aims to evaluate the therapeutic efficacy of a combined treatment approach using conventional occlusion therapy and the home-based PL platform in patients with persistent amblyopia, including those with pathological forms.

## 2. Materials and Methods

### 2.1. Study Design

**Figure 1:**
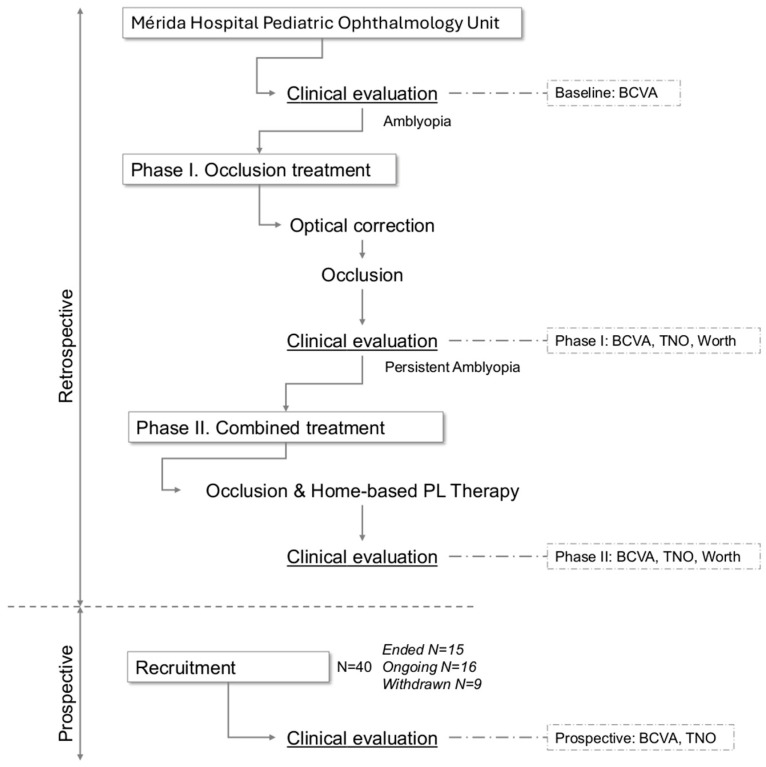
Phases of treatment. The graph represents the two phases of the treatment, marked between the first retrospective clinical evaluation at Mérida Hospital and the prospective clinical evaluation, during which patients were recruited and signed informed consent. The clinical evaluations and outcome measures used in the study are indicated (BCVA: best corrected visual acuity; TNO: stereoacuity test; and Worth 4 dot test). The number of participants (*N*) is also shown.

This was a longitudinal case series of pediatric patients with persistent amblyopia treated at the Ophthalmology Department of Mérida Hospital (Spain) between 2022 and 2025. Outcomes were assessed at four key time points (Figure 1): (a) baseline; (b) plateau after Phase I occlusion treatment; (c) during Phase II combined treatment, if completed; and (d) at a prospective evaluation visit. Data for time points (a), (b), and (c) were collected retrospectively from medical records, whereas time point (d) was assessed prospectively.

### 2.2. Recruitment Procedure

Eligible patients were identified from medical records and contacted to participate in the study. Participants were recruited by telephone through the Ophthalmology Department of Mérida Hospital (Mérida, Extremadura, Spain). They were then invited to the hospital. The informed consent and prospective evaluation were on the same day, which included BCVA and stereoacuity testing.

### 2.3. Participants

Eligible patients had to meet both clinical and methodological criteria. From a methodological standpoint, participants were required to have received the Visionary software as part of their prescribed treatment. From a clinical standpoint, they had to have been diagnosed with patch-resistant amblyopia, including anisometropic, strabismic, combined mechanism (strabismus and anisometropia), and congenital pathological amblyopia. Anisometropia was defined as a difference of 1 or more diopters in spherical equivalent [28]. Strabismic amblyopia was defined if strabismus was observed with unilateral cover test in distance, near, or both. Congenital pathological amblyopia was defined as amblyopia caused by any condition obstructing the visual pathway [7]. Bilateral amblyopia was defined when both eyes had a BCVA ≥ 0.20 logMAR (0.63 decimal) [29].

A patient was considered to have persistent amblyopia if, despite prior treatment with full optical correction (for two months) followed by occlusion or penalization of the fellow eye for more than 32 weeks (in mild cases) or 48 weeks (in severe cases) [6], the interocular difference in BCVA remained ≥ 2 logMAR lines and the amblyopic eye had a BCVA worse than 0.10 logMAR (equivalent to 0.8 decimal acuity) [27]. The prescribed occlusion regimen was two hours per day for mild to moderate amblyopia and six hours per day for severe amblyopia, administered seven days per week [4]. Mild amblyopia was classified as being logMAR BCVA visual acuity of 0.13 to 0.30, moderate amblyopia as being worse than 0.30 to 0.70, and severe amblyopia as being worse than 0.70 [30]. Although subjects with bilateral pathological amblyopia did not receive occlusion treatment, they are included in the persistent amblyopia group. Patients with cognitive delay were excluded from the study.

The study included 40 patients (16 males and 24 females) with a mean age of 9.4 ± 3.4 years. Among them, 17 participants (42%) had anisometropic amblyopia, 4 (10%) had strabismic amblyopia, and 7 (18%) were diagnosed with combined amblyopia. In addition, 12 participants (30%) presented with pathological amblyopia, of whom 7 had deprivation amblyopia, six due to cataracts (two bilateral) and one due to lens subluxation. The characteristic of each participant is shown in Appendix A Table A1.

All participants underwent occlusion treatment for more than 32 weeks with a median of 48.00 ± 21.48 weeks (95% CI, 41.13–54.87 weeks) of prescribed occlusion except participants with bilateral deprivation amblyopia that were not occluded. The home-based PL platform (Visionary) was prescribed once BCVA had stabilized, defined as no change across at least two consecutive ophthalmologic visits.

### 2.4. Clinical Evaluation

BCVA was assessed using a logMAR chart (VistaVision Far Vision Device, Torino, Italy). The number of letters correctly read was recorded, with each letter corresponding to 0.02 logMAR units. Final scores were calculated accordingly [31].

The Simultaneous Prism and Cover Test was used to measure tropia at distance (4 m) and near (1/3 m or 33 cm) with an accommodative target (an isolated letter 2 lines larger than the BCVA of the amblyopic eye).

Refractive error was determined using an automated refractor (Nidek ARK-1, Gamagori Aichi, Japan) and/or retinoscopy under cycloplegia, following Pediatric Eye Disease Investigator Group (PEDIG) guidelines [4]. Cycloplegia was induced with two drops of 1% cyclopentolate administered five minutes apart, and measurements were taken 45 min after the final instillation. In addition, a comprehensive evaluation of the anterior and posterior segments was performed.

Binocular vision (fusion, suppression, or diplopia) was evaluated at four meters, using the Worth Four Dot test on a screen (VistaVision Far Vision Device, Torino, Italy). Stereoacuity was assessed using the TNO stereotest (Lameris Ootech BV, Ede, The Netherlands), a global stereopsis test based on random-dot anaglyphs and administered under standardized lighting conditions using red-green glasses. The test evaluates stereoacuity in discrete steps of 480, 240, 120, 60, 30, and 15 arcseconds. Plates III, IV, and V are considered qualitative pretest plates and are not assigned a specific disparity value by the manufacturer. However, for the purpose of statistical analysis, a value of 1200 arcseconds was arbitrarily assigned to participants who correctly identified these plates but failed to detect the 480 arcsecond plate [27]. An arbitrary value of 1300 arcseconds, was assigned to participants with no measurable stereoacuity (i.e., those unable to identify any stereoscopic figures) [27].

### 2.5. Phases of Treatment

Prior to initiating occlusion therapy, all patients underwent an 8-week period of full optical correction to allow for refractive adaptation. Phase I (occlusion therapy) was then prescribed and continued until BCVA reached a plateau (32 weeks in mild cases and 48 weeks in severe cases). Phase II (combined occlusion plus home-based PL training) was initiated immediately after patients reached this plateau. During Phases I and II, patients were monitored at routine ophthalmology visits approximately every four months in Phase I and two months in Phase II, where BCVA and binocular vision were assessed to guide clinical management.

In addition to these routine visits, a single prospective evaluation visit was scheduled, which included patients who had either completed treatment (Ended group), were still undergoing combined therapy (Ongoing group), or had discontinued prematurely (Withdrawn group).

Visionary is an EU-certified Class I medical device (MDR 2017/745) designed for amblyopia treatment. It is designed for Windows computers with an internet connection. The home-based PL platform includes five adaptive gamified exercises using dichoptic Gabor stimuli (Frisbee Hunt, Balloon Buster, Cookie Crunch, The Big Race, and Crossing the River). Each training session lasted 30 min and involved approximately 500 stimulus–response trials, allowing the estimation of cumulative exposure in terms of both session number and total practice trials.

**Figure 2:**
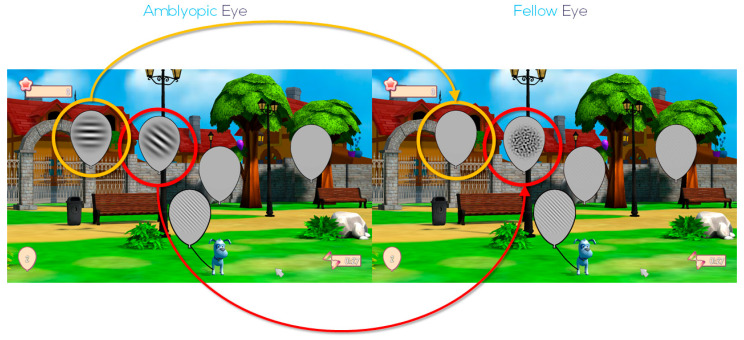
Balloon Buster game. Participants must find and pop balloons showing the same texture (a Gabor patch) as the dog holds. If BCVA is worse than 0.40 logMAR (0.40 decimal), the fellow eye is occluded during the exercise to prevent crosstalk. Otherwise, patients wear anaglyph glasses with the red filter over the amblyopic eye for consistency. The task is to identify the patch’s orientation (horizontal, 45°, vertical, or 135°). The Gabor patch’s size, frequency, and contrast adjust automatically to the patient’s BCVA. When BCVA is worse than 0.20 logMAR (0.63 decimal), the fellow eye receives a medium grey tone stimulus at the patch location (balloon with yellow marker). Once BCVA improves beyond 0.20 logMAR, band-filtered noise matching the patch’s peak spatial frequency is shown to the fellow eye in selected trials (balloon with red marker). Noise contrast is set at the highest tolerable level without affecting contrast sensitivity at that frequency.

Training was monocular (with fellow eye occluded) if BCVA was 0.50 logMAR or worse, and binocular using full refractive correction and red-green anaglyph glasses when BCVA improved above this threshold (Table 1). All exercises use Gabor patches that vary automatically in direction, frequency, and contrast. Once BCVA improves beyond 0.20 logMAR, band-filtered noise matching the patch’s peak spatial frequency is shown to the fellow eye in some trials. The platform automatically records objective compliance data, including the number of completed sessions, cumulative training time, and performance metrics, allowing precise monitoring of adherence to the PL intervention. Patient performance is analyzed by the software to adapt the contrast to the patient’s current threshold for each trained frequency. The software automatically adjusts the frequencies to be trained according to the BCVA values periodically set by the practitioner. One of the gamified exercises, Balloon Buster (Figure 2), is shown as an example.

The typical regimen consisted of 30 min sessions, five days per week. Two participants with pathological amblyopia had bilateral involvement. Treatment was monocular, alternating eyes every 15 days.

Phase II Combined treatment was discontinued once best BCVA in the amblyopic eye improved to better than 0.1 logMAR (equivalent to a decimal acuity greater than 0.8). Patients who did not reach this threshold were encouraged to continue therapy.

Strabismic patients with BCVA equal or worse than 0.20 logMAR were managed according to deviation size and sensory correspondence. The angle of deviation was measured at 4 m using the Simultaneous Prism and Cover Test. Patients with deviations < 12 prism diopters performed the home-based PL training program without prism correction. For deviations ≥ 12 prism diopters, strabismus surgery was recommended. Sensory correspondence was assessed at 4 m using the Worth Four Dot test while neutralizing the deviation with prisms placed over the fellow (dominant) eye. The presence of fusion and full orthotropia under these conditions was considered indicative of normal sensory correspondence. These prisms were worn full-time, and patients performed all home-based PL training while using them until the time of surgery. Patients with anomalous correspondence, who could not fuse under prism correction, performed the home-based PL training without prisms.

### 2.6. Treatment Discontinuation

Phase II was stopped when BCVA in the amblyopic eye reached 0.00 logMAR (1.0 decimal). Patients not reaching this threshold were encouraged to continue.

### 2.7. Prospective Evaluation Visit

Participants were invited to the hospital for a prospective evaluation visit. At this single visit, written informed consent was obtained, and BCVA and stereoacuity were assessed. In patients who had completed treatment, the interval between the last Visionary exercise and the prospective evaluation visit ranged from 0 to 31 months (mean 14.9 months; median 16.5 months). No washout period was implemented, as the study aimed to evaluate visual outcomes under routine clinical conditions.

### 2.8. Statistical Analysis

Data were analyzed using SPSS 26.0 (Chicago, IL, USA). Normality was tested with the Kolmogorov–Smirnov test. Continuous variables are presented as mean ± standard deviation, and non-parametric tests (Wilcoxon rank-sum and Mann–Whitney U) were applied where appropriate.

In both Phase I (occlusion therapy) and Phase II (combined occlusion plus home-based PL training), the main outcome variables were BCVA in the amblyopic eye and stereoacuity. For patients with bilateral amblyopia, analyses were based on the eye with the poorer BCVA. Compliance with spectacle correction and occlusion therapy during Phase I was not systematically monitored and therefore was not included in the analysis.

For Phase II, patients were first described according to treatment status (Ended, Ongoing, and Withdrawn) to illustrate clinical trajectories. However, for the efficacy analysis, a post hoc exploratory analysis was performed to account for adherence to home-based PL training, which provides objective logs of usage.

The home-based PL training platform automatically recorded objective compliance data, including total training hours and number of sessions. For this study, cumulative training hours were used as the primary indicator of compliance. The analysis was conducted at the patient level (*N* = 40), using the worse eye at baseline in bilateral cases. Patients were stratified into two subgroups according to cumulative home-based PL training: <10 h (low exposure) and ≥10 h (higher exposure).

Phase II combined treatment was prescribed until the amblyopic eye achieved a BCVA of 0.00 logMAR. For the purposes of statistical analysis, treatment success was defined as either a gain of ≥2 logMAR lines or a final BCVA of 0.10 logMAR or better. In cases with minimal baseline deficit (e.g., baseline BCVA close to 0.10 logMAR), patients reaching 0.10 logMAR without clinically meaningful improvement were not considered treatment successes, to avoid overestimating treatment effects. An improvement in stereoacuity was considered clinically meaningful when there was a gain of at least two levels on the TNO test, or when a patient with no measurable stereopsis at baseline achieved a stereoacuity of 1200 arcsec or better as measured with the TNO.

Baseline characteristics were compared between subgroups (non-pathologic vs. pathologic; <10 h vs. ≥10 h home-based PL training exposure) using the Mann–Whitney U test for continuous variables. Particular attention was given to assessing comparability of treatment regimens between groups, including prescribed hours of occlusion and cumulative exposure to PL exercises.

Contingency tables were constructed to compare treatment success proportions between exposure groups, using chi-square or Fisher’s exact tests as appropriate. Effect sizes were expressed as relative risk (RR), odds ratio (OR), and absolute risk difference (ARD), each with corresponding 95% confidence intervals (CIs).

To account for potential confounding, multivariable binary logistic regression analyses were performed to identify independent predictors of BCVA recovery and stereoscopic visual acuity recovery. Separate models were constructed for each outcome. Predictor variables were selected a priori based on clinical relevance and included cumulative PL exposure, age at treatment initiation, baseline BCVA of the amblyopic eye, presence of pathological amblyopia, and presence of strabismus. Adjusted odds ratios (ORs) with 95% confidence intervals (CIs) and corresponding *p*-values were calculated. Variables were retained in the final models based on their clinical relevance and contribution to model stability. Statistical significance was defined as *p* < 0.05.

Time-to-event analyses were performed using Kaplan–Meier survival methodology based on individual patient data. The cumulative probability of treatment completion was further modelled using a nonlinear exponential function fitted by least-squares regression. These analyses were performed using MATLAB R2020a (MathWorks, Natick, MA, USA).

Kaplan–Meier analysis was used to evaluate time to treatment success during Phase II, defined as the number of weeks from initiation of PL therapy to achievement of treatment success (≥2 logMAR lines gain or final BCVA ≤ 0.10 logMAR). Patients who did not achieve treatment success were censored at their last follow-up visit, and differences between exposure groups (<10 h vs. ≥10 h) were assessed using the log-rank test.

## 3. Results

A total of 40 patients were included in the study. Baseline descriptive data and individual ocular diagnoses are presented in Appendix A Table A1.

The mean of baseline BCVA in the fellow eye was 0.02 ± 0.08 logMAR units (95% CI, −0.01–0.05). In amblyopic eyes the mean was 0.45 ± 0.23 logMAR (95% CI, 0.37–0.52). In a small subset of cases with bilateral pathological amblyopia, both eyes had BCVA poorer than 0.10 logMAR, which explains why some fellow eyes also met the amblyopia definition.

Baseline characteristics are summarized in Table 2. Groups were broadly comparable in terms of BCVA at baseline. However, patients in the <10 h exposure group were significantly older (*p* = 0.01), and pathologic amblyopia was more frequent in this group (*p* = 0.02). Prescribed occlusion regimens and cumulative exposure to PL were otherwise similar across groups, as expected, with the exception that the <10 h group necessarily had significantly lower training exposure.

### 3.1. Phase I: Occlusion Therapy

**Figure 3:**
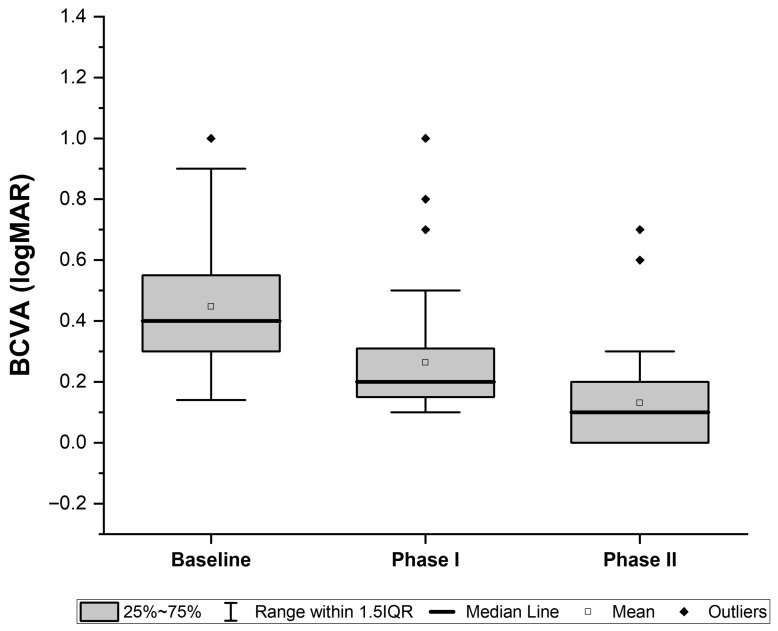
Box-and-whisker plot of best-corrected visual acuity (BCVA, logMAR) at baseline, after Phase I (occlusion therapy), and after Phase II (combined therapy). Boxes represent the interquartile range (IQR, 25th–75th percentiles), horizontal lines indicate the median, and whiskers show values within 1.5× IQR. The square marks the mean, and diamonds represent outliers.

**Figure 4:**
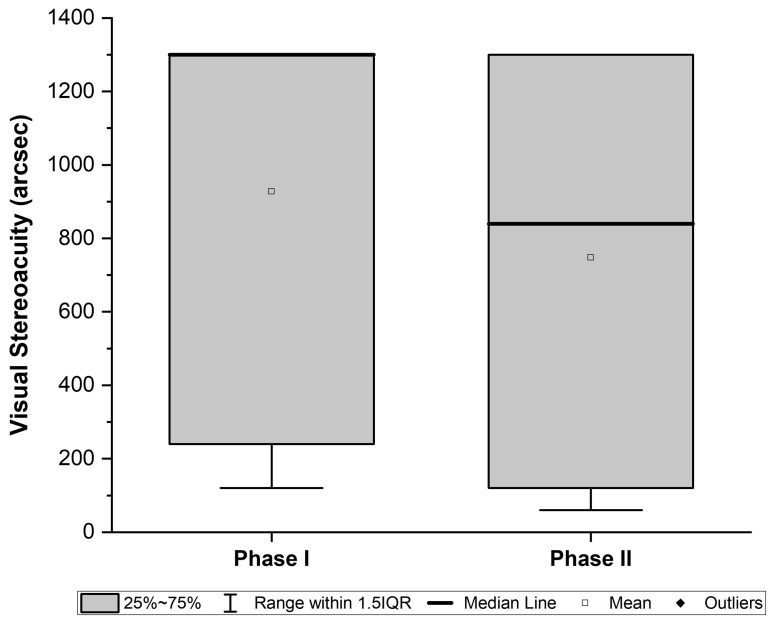
Box-and-whisker plot of stereoacuity (arcsec, TNO test) at the end of Phase I (occlusion therapy) and after Phase II (combined therapy). Boxes represent the interquartile range (IQR, 25th–75th percentiles), horizontal lines indicate the median, and whiskers show values within 1.5× IQR. The square marks the mean, and diamonds represent outliers. In Phase I, the median coincided with the upper quartile, so the line is not visible inside the box.

Following occlusion therapy, BCVA in the amblyopic eye improved significantly from 0.45 ± 0.23 (95% CI, 0.37–0.52) to 0.26± 0.19 logMAR (95% CI, 0.20–0.52) (*p* < 0.01) (Figure 3). Baseline data on binocular vision prior to occlusion therapy was not available. At the end of Phase I, 21 of 40 subjects demonstrated suppression on the Worth test, and 23 had no measurable stereoacuity (Figure 4).

Non-pathologic amblyopia (*N* = 28): BCVA improved from 0.41 ± 0.19 (95% CI, 0.34–0.49) to 0.20 ± 0.09 logMAR (95% CI, 0.17–0.24) (*p* < 0.01).

Pathologic amblyopia (*N* = 12): BCVA improved from 0.53 ± 0.30 logMAR (95% CI, 0.34–0.72) to 0.41 ± 0.28 logMAR (95% CI, 0.23–0.59) (*p* < 0.05). Within the pathologic amblyopia group, seven patients had congenital cataract (deprivation amblyopia). In this subgroup, BCVA improved from 0.35 ± 0.13 logMAR (95% CI, 0.23–0.47) to 0.20 ± 0.08 logMAR (95% CI, 0.17–0.24) (*p* = 0.04).

### 3.2. Phase II: Combined Therapy

With the addition of home-based PL, further gains were observed. Overall, BCVA improved from 0.26 ± 0.19 (95% CI, 0.20–0.52) to 0.13 ± 0.16 logMAR (95% CI, 0.08–0.18) (*p* < 0.01) (Figure 3) and stereoacuity improved from 928 ± 505 arc sec (95% CI, 766–1089) to 748 ± 558 arcsec (95% CI, 569–92) (*p* = 0.01). Fusion on the Worth Four Dot test was restored in 11 of 19 patients (58%) who had suppression after Phase I. In addition, six patients with no measurable stereoacuity at baseline achieved stereopsis during Phase II (Figure 4).

Non-pathologic amblyopia: BCVA improved from 0.20 ± 0.09 logMAR (95% CI, 0.17–0.24) to 0.09 ± 0.01 logMAR (95% CI, 0.05–0.13) (*p* < 0.01). Regarding binocular vision, nine patients achieved fusion, and five who had no measurable stereoacuity at baseline acquired measurable stereoacuity. Stereoacuity improved from 949 ± 501 arcsec (95% CI, 754–1142) to 685 ± 545 arcsec (95% CI, 474–896) (*p* = 0.02).

Pathologic amblyopia: BCVA improved from 0.41 ± 0.19 logMAR (95% CI, 0.34–0.49) to 0.22 ± 0.23 logMAR (95% CI, 0.08–0.37) (*p* < 0.05). In terms of binocular vision, no patients achieved fusion on the Worth test, and stereoacuity showed no improvement (*p* = 1.00). In deprivation amblyopia, BCVA improved from 0.20 ± 0.08 logMAR (95% CI, 0.17–0.24) to 0.07 ± 0.08 logMAR (95% CI, 0.04–0.11) (*p* = 0.03). Prior to the combined therapy, one patient exhibited suppression and was unable to achieve fusion, and three patients had no measurable stereoacuity. None of these individuals demonstrated improvement in binocular vision, and the observed changes in stereoacuity were not statistically significant (*p* = 0.27).

Eleven patients presented with strabismus (6 esotropia, 3 exotropia, 2 hypertropia; mean baseline deviation 17.2 ± 11.5 prism diopters (95% CI, 9.5–24.9). Five esotropic patients underwent surgery, reducing their deviation from 28.0 ± 5.7 prism diopters (95% CI, 22.8–33.2) to 1.6 ± 2.6 prism diopters (95% CI –0.9 to 4.1). All achieved fusion postoperatively, with three recovering measurable stereoacuity (240–1200 arcsec). Among the 35 non-surgical patients, mean stereoacuity improved from 896 ± 517 arcsec (95% CI, 716–1076) to 646 ± 576 arcsec (95% CI, 452–841) (*p* < 0.05), and the proportion of participants demonstrating fusion on the Worth Four Dot test increased from 40.0% to 74.0%.

### 3.3. Treatment Completion, Ongoing Therapy, and Withdrawal

To better illustrate patient trajectories during Phase II, participants were classified according to their treatment status at the time of analysis. This categorization highlights the proportion of patients who successfully completed therapy, those who remained in active treatment, and those who discontinued home-based PL training prematurely.

**Figure 5:**
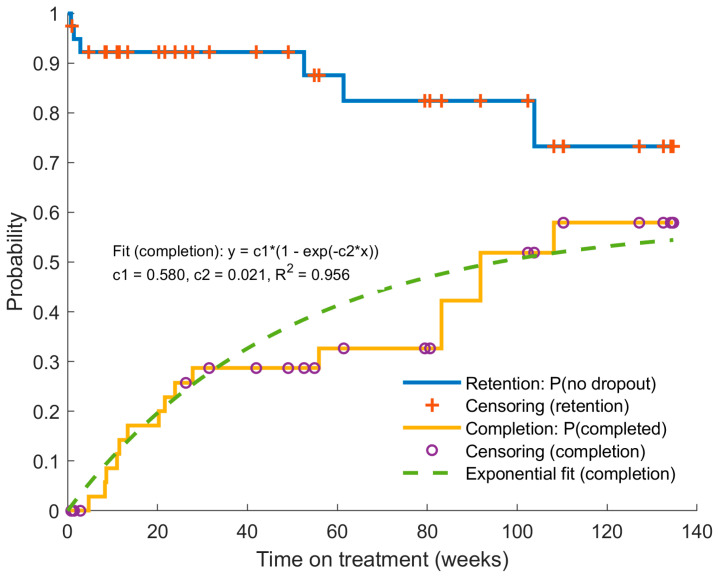
Kaplan–Meier time-to-event analyses for treatment withdrawal and treatment completion during Phase II therapy. The X-axis represents weeks of treatment until the prospective evaluation visit; the Y-axis shows the estimated probability. One curve represents the probability of remaining free from withdrawal over time, and the other represents the cumulative probability of treatment completion. An exponential model was fitted to the completion curve, allowing estimation of the time required to reach 90% of the asymptotic completion probability.

At the time of analysis, 15 patients had achieved a final BCVA better than 0.1 logMAR (equivalent to a decimal acuity greater than 0.8) and were no longer receiving therapy. Sixteen patients were still undergoing combined treatment (occlusion plus home-based PL training), while nine patients had withdrawn prematurely and continued with occlusion therapy alone. The mean duration of Phase II therapy was 38.7 ± 34.1 weeks in the Ended group, 76.2 ± 40.1 weeks in the Ongoing group, and 37.4 ± 35.9 weeks in the Withdrawn group. Figure 5 shows the Kaplan–Meier estimates for time to withdrawal and time to treatment completion. For the analysis of treatment withdrawal, patients in the Withdrawn Group were defined as events, whereas patients in the Ended and Ongoing Groups were treated as censored observations. For the analysis of treatment completion, patients in the Ended Group were defined as events, and those in the Withdrawn and Ongoing Groups were treated as censored observations. The cumulative probability of treatment completion was well described by an exponential model, with 90% of the estimated asymptotic completion probability reached after 109.65 weeks (25.58 months). The probability of remaining free from withdrawal remained above 73.25% at 130 weeks, indicating sustained long-term adherence.

Moreover, at the prospective evaluation visit, both BCVA and stereoacuity remained stable in the Ended group, even in patients with follow-up intervals exceeding 24 months (*p* < 0.01 for both outcomes).

These trajectories provide important context, but efficacy outcomes were ultimately examined according to cumulative exposure to home-based PL training (<10 h vs. ≥10 h), which allowed a more objective assessment of treatment compliance.

### 3.4. Exposure–Response Analysis (Phase II)

To explore whether treatment outcomes varied with cumulative home-based PL training, patients were stratified into <10 h (low exposure, *N* = 15) and ≥10 h (higher exposure, *N* = 25) subgroups.

BCVA treatment success defined as a gain of ≥2 logMAR lines or a final BCVA ≤ 0.10 logMAR in the amblyopic eye—was achieved in 72.0% (18/25) of the ≥10 h group compared with 40.0% (6/15) of the <10 h group. This difference was statistically significant (χ^2^(1) = 5.99, *p* = 0.05; Fisher’s exact *p* = 0.021). The relative risk of success in the ≥10 h group was 1.94 (95% CI, 1.01–3.73), the odds ratio was 5.25 (95% CI, 1.33–20.76), and the absolute risk difference was +37.8% (95% CI, +8.4% to +67.1%).

The clinically meaningful stereoacuity defined as a gain ≥ 2 levels or, in patients with no measurable stereopsis at baseline, achieving a stereoacuity of 1200 arcsec or better, was achieved in 40.0% (10/25) of the ≥10 h group compared with 20.0% (3/15) of the <10 h group. Although not statistically significant (χ^2^(1) = 1.37, *p* = 0.24; Fisher’s exact *p* = 0.27), the effect estimates favored the ≥10 h group (RR = 2.00, 95% CI, 0.63–6.36; OR = 2.67, 95% CI, 0.61–11.67; ARD = +20%, 95% CI, –11% to +51%).

### 3.5. Multivariable Analysis of Predictors of Visual and Stereoscopic Recovery

To account for potential confounding and identify independent predictors of treatment outcomes, multivariable logistic regression analyses were performed for both BCVA recovery and stereoscopic visual acuity recovery.

For BCVA recovery, cumulative PL exposure remained independently associated with treatment success after adjustment for age, baseline BCVA, and presence of pathological amblyopia (adjusted OR = 4.98, 95% CI 1.14–21.74, *p* = 0.03). This finding indicates that greater PL exposure significantly increased the likelihood of visual improvement. Other variables, including baseline BCVA, pathological amblyopia, and presence of strabismus, were not independently associated with BCVA recovery in the adjusted model.

For stereoscopic visual acuity recovery, cumulative PL exposure was also independently associated with improvement (OR = 6.95, *p* = 0.04). Baseline BCVA was a strong independent predictor (OR = 0.00, *p* = 0.02), with worse initial visual acuity associated with lower probability of stereopsis recovery. The presence of pathological amblyopia and strabismus did not reach statistical significance but was retained in the model due to their clinical relevance and potential confounding effects.

## 4. Discussion

This study evaluated the efficacy of a two-phase protocol for the treatment of persistent amblyopia, combining conventional occlusion therapy with home-based PL using the Visionary program. The results suggest that this combined approach leads to significant improvements in monocular visual acuity, including in patients with amblyopia associated with congenital ocular pathology. However, improvements in binocular function were primarily observed in non-pathologic cases, while patients with pathological amblyopia showed limited binocular recovery.

Following occlusion therapy alone (Phase I), BCVA improved significantly from 0.45± 0.23 logMAR to 0.26 ± 0.19 logMAR (*p* < 0.01), consistent with previously reported outcomes [4]. However, previous studies have shown that visual gains tend to plateau with extended occlusion [6]. In our cohort, the subsequent addition of PL during Phase II led to further and clinically meaningful improvement in BCVA, with final values reaching 0.13 ± 0.16 logMAR on average suggesting a therapeutic benefit beyond conventional occlusion, and consistent with results from prior home-based PL training studies [26,27].

Although mild amblyopia is conventionally defined up to 0.30 logMAR, in our study patients were included if their amblyopic eye remained worse than 0.10 logMAR after prolonged occlusion therapy. This stricter threshold was applied to ensure that even those with relatively mild residual deficits, but still clinically relevant interocular differences, were captured.

The exploratory analysis stratified by home-based PL training time provides additional insight into treatment outcomes. Eyes completing ≥ 10 h of training were nearly twice as likely to achieve success as those with <10 h (77.8% vs. 40.0%; RR = 1.94, 95% CI: 1.01–3.73; Fisher’s exact *p* = 0.02), suggesting a potential dose response effect. Importantly, home-based PL training therapy was uniformly prescribed by the ophthalmologist, and differences in exposure reflected practical barriers to adherence (e.g., outdated computers, poor internet access, reliance on relatives’ devices, or refusal of the child to participate).

Patients in the <10 h group were older and more often had pathologic amblyopia, factors likely influencing outcomes. Even so, they still showed meaningful BCVA gains, possibly because poor compliance with digital exercises indirectly favored better adherence to patching. This highlights the complex interplay between treatment components and the need for objective monitoring of patching compliance in future studies. Patients in the <10 h group were older and more frequently had pathological amblyopia, factors that may have influenced treatment outcomes. Multivariable analysis demonstrated that higher perceptual learning exposure was independently associated with significantly increased odds of both BCVA and stereoscopic visual acuity recovery, supporting a clinically meaningful exposure–response relationship. Interestingly, the presence of strabismus was not independently associated with stereopsis recovery in the adjusted model. This finding should be interpreted cautiously, as the limited sample size may have reduced statistical power, and strabismus remains an important prognostic factor in clinical practice. These results reflect real-world variability in treatment adherence and clinical characteristics. While promising, the analysis was post hoc and limited by sample size, precluding definitive conclusions regarding causality. Nevertheless, the findings support the hypothesis that greater cumulative perceptual learning exposure contributes meaningfully to visual recovery and underscore the need for prospective, controlled studies to establish optimal treatment dosage.

In terms of binocular function, participants demonstrated significant gains in stereoacuity, improving from 949 ± 501 arcsec to 685 ± 545 arcsec (*p* = 0.02). Fusion rates on the Worth Four Dot test increased from 52% at baseline to 88% post-treatment. These results align with earlier studies using dichoptic Gabor stimuli [32], highlighting the potential of PL not only to improve monocular function, but to enhance binocular integration as well. The outcomes observed in patients with strabismus are particularly noteworthy. Five of them underwent strabismus surgery. Prior to surgery, all had continuous Fresnel prism correction of their strabismus. Postoperatively, they demonstrated fusion on the Worth 4-dot test, and three of them achieved measurable stereoacuity, assessed without the use of prisms. These findings are consistent with previous studies [27,33].

Outcomes in patients with amblyopia associated with ocular pathology were less consistent than in those with non-pathologic forms. At baseline, the pathologic group started with a mean BCVA of 0.53 ± 0.30 logMAR, which improved to 0.41 ± 0.28 logMAR after occlusion therapy (Phase I) and further to 0.22 ± 0.23 logMAR at the prospective evaluation following combined therapy (Phase II) (overall *p* < 0.05). Despite these visual gains, stereoacuity outcomes remained poor, and no patients achieved fusion on the Worth test. Within this group, patients with deprivation amblyopia due to congenital cataract demonstrated a greater potential for recovery, with BCVA improving from 0.45 ± 0.27 logMAR at baseline to 0.07 ± 0.08 logMAR after Phase II combined therapy. However, these patients did not achieve measurable binocular vision, and stereoacuity outcomes remained unchanged, with none reaching a clinically meaningful gain. In contrast, patients with non-deprivation pathology such as coloboma, glaucoma, or foveal hypoplasia showed even more limited responses, reflecting the impact of structural anomalies on visual prognosis. These findings underscore the importance of distinguishing deprivation from non-deprivation forms of pathologic amblyopia, as their recovery potential and responsiveness to therapy are markedly different.

Treatment timelines revealed a clear distinction across subgroups. The mean duration of Phase II therapy was 38.7 ± 34.1 weeks in the Ended group, 76.2 ± 40.1 weeks in the Ongoing group, and 37.4 ± 35.9 weeks in the Withdrawn group. An exponential trend was observed in the cumulative probability of treatment completion: 90% of the estimated asymptotic completion probability was reached at approximately 109.7 weeks (25.6 months) (Figure 5). Most patients (two-thirds) who withdrew did so at the beginning rather than at later stages. These findings suggest that meaningful recovery, particularly in more complex cases, may require extended treatment periods beyond those typically associated with occlusion alone^5^. This is likely due to the gradual neural changes associated with PL, which promotes reweighting of cortical responses and binocular integration [34,35].

At the prospective evaluation visit, both visual acuity and stereoacuity remained stable in patients from the Ended group, including those with and without associated ocular pathology. The treatment duration (mean 38.7 ± 34.1 weeks), involving thousands of practice trials distributed across multiple sessions, may have contributed to the stability of these outcomes [15,16,17].

Finally, the study limitations must be acknowledged. The relatively small sample size may limit the generalizability of the findings and reduce statistical power for subgroup analyses. The lack of a control group and examiner masking limits causal inference. Additionally, differences in age and prevalence of pathological amblyopia between training exposure groups may have confounded the observed association between PL exposure and treatment success. Therefore, the exposure–response findings should be interpreted with caution. Furthermore, the combination of retrospective data collection and a single prospective evaluation visit may introduce selection bias, as only patients who attended the prospective evaluation were included. This may have resulted in overrepresentation of more compliant patients. Compliance with spectacles and occlusion therapy during both Phase I and Phase II was not objectively monitored, which may have influenced outcomes. In contrast, compliance with the home-based PL training program could be quantified, allowing for the exploratory exposure–response analysis. Without objective compliance data for conventional treatments, however, the independent contribution of PL cannot be definitively isolated. Moreover, treatment success may partly reflect overall adherence rather than the independent effect of the PL component alone, as patients who completed more training sessions may have also been more adherent to occlusion therapy and follow-up recommendations. Future studies incorporating objective monitoring of occlusion compliance would help clarify the independent contribution of each treatment component.

## 5. Conclusions

Combined occlusion therapy and gamified home-based PL improved visual outcomes in children with persistent amblyopia, with meaningful gains in BCVA and more modest improvements in stereoacuity, particularly in non-pathologic cases. A post hoc exposure response analysis showed that patients with higher cumulative training (≥10 h) achieved greater improvements, underscoring the importance of compliance. Although the lack of a formal control group limits causal inference, these findings suggest that gamified home-based PL may represent a useful adjunct to standard occlusion therapy and highlight the need for future controlled studies.

## Figures and Tables

**Table 1:** Visionary VA exercises configuration: BCVA of amblyopic eye; Gabor patch size; Gabor patch frequency; patch/anaglyph glasses; and band-filtered noise.

BCVA	Gabor Size	Frequency	Patch/Anaglyph	Band-Filtered Noise
1.10	5.0	1; 2	Patch	No
1.00	5.0	1; 2	Patch	No
0.90	5.0	1; 2; 4	Patch	No
0.80	5.0	1; 2; 4	Patch	No
0.70	5.0	1; 2; 4	Patch	No
0.60	5.0	1; 2; 4	Patch	No
0.50	3.0	2; 4; 8	Patch	No
0.40	3.0	2; 4; 8	Anaglyph	No
0.30	2.0	4; 8; 12	Anaglyph	No
0.20	1.5	8; 12; 16	Anaglyph	Yes
0.10	1.0	12; 16; 24	Anaglyph	Yes
0.00	1.0	12; 16; 24	Anaglyph	Yes

Abbreviations: BCVA = best corrected visual acuity. Units: BCVA, LogMAR; Gabor size, degrees; Frequency, cycles per degree.

**Table 2:** Baseline characteristics grouped by home-based perceptual learning platform (Visionary) exposure and presence or not of pathology.

	Low Exposure < 10 h (*N* = 15)		Higher Exposure ≥ 10 h (*N* = 25)		*p*
Age (Years)	10.89 ± 4.20	(8.56~13.21)	8.18 ± 2.34	(7.22~9.14)	0.02
Refraction FE (D)	1.41 ± 1.79	(0.42~2.40)	0.40 ± 2.55	(−0.65~1.45)	0.12
Refraction AE (D)	2.73 ± 2.41	(1.39~4.06)	0.11 ± 5.18	(−2.03~2.25)	0.15
BCVA FE (logMAR)	0.00 ± 0.00	(−0.06~0.08)	0.03 ± 0.05	(0.01~0.05)	0.89
BCVA AE (logMAR)	0.39 ± 0.21	(0.28~0.51)	0.48 ± 0.24	(0.38~0.58)	0.25
Occlusion (Weeks)	54.80 ± 24.56	(41.20~68.40)	43.92 ± 18.75	(36.18~51.66)	0.52
PL (Hours)	3.43 ± 3.39	(1.55~5.31)	39.02 ± 25.40	(28.53~49.51)	<0.01
	**Pathology (** ***N*** **= 12)**		**No-Pathology (** ***N*** **= 28)**		***p***
Age (Years)	7.57 ± 2.40	(6.04~9.09)	9.89 ± 3.54	(8.52~11.27)	0.02
Refraction FE (D)	(0.33 ± 2.68)	(−1.37~2.03)	0.97 ± 2.18	(0.12~1.81)	0.48
Refraction AE (D)	0.10 ± 5.09	(−3.13~3.34)	1.52 ± 4.25	(−0.13~3.17)	0.22
BCVA FE (logMAR)	0.07 ± 0.10	(0.00~0.13)	0.00 ± 0.06	(−0.03~0.03)	0.15
BCVA AE (logMAR)	0.53 ± 0.30	(0.34~0.72)	0.41 ± 0.19	(0.34~0.49)	0.36
Occlusion (Weeks)	42.33 ± 23.45	(27.44~57.23)	50.43 ± 20.54	(42.46~58.39)	0.92
PL (Hours)	22.58 ± 13.94	(13.73~31.44)	27.00 ± 30.56	(15.15~38.85)	0.65

Abbreviations: FE = fellow eye; AE = amblyopic eye; BCVA = best corrected visual acuity. PL = Perceptual Learning. Values are presented as mean ± SD with 95% confidence intervals (95% CI). Group comparisons were performed using the Mann–Whitney U test.

## Data Availability

The original contributions presented in this study are included in the article. Further inquiries can be directed to the corresponding author.

## Abbreviations

The following abbreviations are used in this manuscript:

PL	Perceptual Learning
BCVA	Best-Corrected Visual Acuity
FE	Fellow Eye
AE	Amblyopic Eye

## Appendix A

**Table A1:** Descriptive data.

N	Sex	Rx DE	Rx AE	AE	BCVA FE	BCVA AE	Mechanism	Strabismus	Group	Visionary Exposure	Age Patch (Phase I)	Age Visionary (Phase II)
Without ocular pathology												
1	F	0.50	+4.00	LE	0.00	0.48	ANISO		Ongoing	Low	7	12
2	M	−0.25	1.00	LE	0.00	0.20	ANISO		Withdrawn	Low	4	12
3	F	0.75	2.25	RE	0.00	0.70	ANISO		Withdrawn	Low	6	12
4	F	0.25	5.12	LE	0.00	0.48	ANISO		Ended	Higher	7	9
5	F	0.00	0.38	RE	0.00	0.16	ANISO		Ended	Low	6	8
6	M	0.25	3.75	LE	0.00	0.48	ANISO		Ongoing	Higher	5	7
7	F	0.25	5.00	RE	0.00	0.16	ANISO		Ongoing	Higher	7	11
8	F	3.75	3.50	RE	0.00	0.70	STRAB	30 ET (Surgery)	Ongoing	Higher	4	6
9	M	4.62	5.75	RE	0.10	0.30	COMBINED	35 ET (Surgery)	Ended	Higher	3	8
10	F	0.75	1.87	LE	0.00	0.4	ANISO		Withdrawn	Low	5	23
11	M	0.25	1.75	LE	0.00	0.48	ANISO		Withdrawn	Low	6	15
12	M	1.00	1.75	RE	0.00	0.16	ANISO		Ended	Low	8	10
13	M	4.25	5.75	RE	0.00	0.30	COMBINED	25 ET (Surgery)	Withdrawn	Higher	4	6
14	F	0.62	0.25	LE	0.00	0.62	STRAB	2 ET	Withdrawn	Low	5	7
15	M	1.63	5.50	LE	0.00	0.40	ANISO		Withdrawn	Low	2	10
16	F	−0.38	0.25	RE	0.00	0.48	STRAB	4 ET 10 HYPER	Ongoing	Higher	5	10
17	M	2.62	3.50	RE	0.00	0.40	ANISO		Ended	Higher	5	8
18	M	−1.25	−2.88	LE	0.00	0.20	ANISO		Ended	Higher	3	5
19	F	−5.63	−12.88	LE	0.00	0.30	COMBINED	10 XT	Ongoing	Higher	8	14
20	F	−1.25	−7.25	RE	0.1	0.70	ANISO		Ended	Higher	5	7
21	M	2.75	4.12	LE	0.1	0.48	ANISO		Ongoing	Higher	5	7
22	M	3.87	5.62	LE	0.1	0.20	COMBINED	8 HYPER	Ended	Low	4	6
23	F	−0.38	−1.50	RE	0.00	1.00	ANISO		Ongoing	Higher	5	10
24	F	1.25	0.62	LE	0.00	0.48	ANISO		Ended	Higher	5	7
25	F	−0.125	0.63	RE	0.00	0.70	STRAB	15 XT	Ended	Low	5	9
26	M	2.75	3.62	LE	0.00	0.20	STRAB	20 ET (Surgery)	Ended	Higher	5	11
27	M	4.625	5.13	RE	0.00	0.70	ANISO		Ongoing	Low	4	6
28	F	−0.38	−4.13	LE	0.00	0.48	COMBINED	30 ET (Surgery)	Ongoing	Higher	6	9
With ocular pathology												
29	F	0.38	−3.13	RE	0.10	1.00	chorioretinal coloboma		Ongoing	Higher	2	4
30	F	−0.25	−0.13	LE	0.00	0.30	foveal hypoplasia	4 XT	Ongoing	Low	5	9
31	M	−0.125	1.38	RE	0.00	0.40	unilateral CC		Ended	Higher	3	5
32	M	1.50	1.25	RE	0.10	0.20	bilateral CC		Ended	Higher	-	7
33	F	2.75	5.87	LE	0.10	0.20	unilateral CC		Ended	Higher	7	9
34	F	−0.88	1.25	RE	0.00	0.48	unilateral CC		Ended	Higher	4	6
35	F	5.00	8.00	LE	0.10	0.30	lens subluxation		Ongoing	Low	6	13
36	F	−1.13	−0.25	RE	0.2	0.30	unilateral CC		Ongoing	Higher	5	7
37	F	−6.00	−4.00	RE	0.2	0.30	bilateral sutural CC		Ongoing	Higher	-	6
38	F	2.75	2.88	LE	0.3	1.00	chorioretinal coloboma		Withdrawn	Low	4	7
39	M	−0.13	−12.13	LE	0.00	0.70	myelinated fibers macula		Withdrawn	Higher	2	6
40	F	0.38	0.00	LE	0.00	1.20	congenital glaucoma		Ongoing	Higher	4	10

Abbreviations: M, male; F, female; Rx, refraction; FE, fellow eye; AE, amblyopic eye; BCVA, best corrected visual acuity; ANISO, anisometropía; STRAB, strabismus; COMBINED, anisometropia and strabismus combined; ET, esotropia, XT, exotropia; HYPER, hypertropia; CC, congenital cataract. Units: age when patching (phase I) and home-based perceptual learning training (Visionary) (phase II), in years; visual acuity in logarithmic scale and strabismus deviation in prism diopters in baseline (prior occlusion treatment). Patients 32 and 37 with bilateral cataracts were not prescribed patching; therefore, age is not reported here.
